# Two-stage malignant transformation in hamster embryo cells

**DOI:** 10.1038/bjc.1979.2

**Published:** 1979-01

**Authors:** J. A. Poiley, R. Raineri, R. J. Pienta

## Abstract

Transformation of primary hamster embryo cells was investigated using 3-methylcholanthrene (MCA), a combination of MCA and 12-O-tetradecanoylphorbol-13-acetate (TPA), and initiation with MCA or dibenz(a,h)anthracene (DBA) followed by promotion with TPA. Evidence for transformation was (a) abnormal cellular morphology, (b) increased lifespan, (c) growth in soft agar, and (d) tumour induction by s.c. inoculation into suckling hamsters.

Cells treated with either MCA or MCA+TPA showed the same latent period to morphological transformation, although their tumorigenic potential varied. Cells did not form tumours when TPA was administered 7 days after treatment with either MCA or DBA. However, when administration of TPA was delayed to 27 days after treatment with a transforming dose of MCA or a subthreshold dose of DBA, the cells transformed and produced tumours in hamsters.

Our results show that TPA may act as an inhibitor or promoter, depending on the length of time between treatment of the hamster embryo cells with the carcinogen and administration of the TPA. It appears that treatment of cells with TPA before the initiating event is complete inhibits or delays the development of their ability to induce tumours in animals or grow in soft agar. However, with a sufficient interval between the application of the initiating carcinogen and the promoter, transformation occurs, and the ability of cells treated with subthreshold doses of DBA to form tumours is enhanced.


					
Br. J. Cancer (1979) 39, 8

TWO-STAGE MALIGNANT TRANSFORMATION

IN HAMSTER EMBRYO CELLS

IJ. A. POILEY*, R. RAINERI AND R. J. PIENTA

From the (hemtical Carcinogenesis Program, NCJ, Frederick Cancer Research Center,

Frederick, Maryland 21701, U.S.A.

Received 24 February 1978 Accepted 3 October 1978

Summary.-Transformation of primary hamster embryo cells was investigated
using 3-methylcholanthrene (MCA), a combination of MCA and 12-0-tetradecanoyl-
phorbol-13-acetate (TPA), and initiation with MCA or dibenz(a,h)anthracene
(DBA) followed by promotion with TPA. Evidence for transformation was
(a) abnormal cellular morphology, (b) increased lifespan, (c) growth in soft agar,
and (d) tumour induction by s.c. inoculation into suckling hamsters.

Cells treated with either MCA or MCA+TPA showed the same latent period to
morphological transformation, although their tumorigenic potential varied. Cells
did not form tumours when TPA was administered 7 days after treatment with
either MCA or DBA. However, when administration of TPA was delayed to 27 days
after treatment with a transforming dose of MCA or a subthreshold dose of DBA,
the cells transformed and produced tumours in hamsters.

Our results show that TPA may act as an inhibitor or promoter, depending on
the length of time between treatment of the hamster embryo cells with the
carcinogen and administration of the TPA. It appears that treatment of cells with
TPA before the initiating event is complete inhibits or delays the development of
their ability to induce tumours in animals or grow in soft agar. However, with a
sufficient interval between the application of the initiating carcinogen and the
promoter, transformation occurs, and the ability of cells treated with subthreshold
doses of DBA to form tumours is enhanced.

THE ABILITY of promoting agents to
enhance the formation of tumours in
mouse skin by carcinogens has been re-
viewed by Boutwell (1964, 1974). In vitro
effects of 12-0-tetradecanoyl-phorbol- 13-
acetate (TPA) on cellular multiplication,
RNA synthesis and DNA repair synthesis
have been studied in mouse fibroblasts
(Sivak & Van Duuren, 1970; Sivak et al.,
1969, 1972; Trosko et al., 1975). More
recently, TPA has been shown to en-
hance the focal transformation rate of
mouse C3H/1OT1/2 cells (Mondal & Heidel-
berger, 1976; Mondal et al., 1976) and to
enhance transformation in rat fibroblasts
in culture (Lasne et al., 1974, 1977). The
rate of focal transformation in mouse cells
could be increased or decreased, depending

* To whom requests for reprints should be sent.

on the time at which TPA was admini-
stered relative to the carcinogen (Mondal
et al., 1976). Our studies were undertaken
to determine whether application of the
promoting agent, TPA, would reduce the
time from treatment with a carcinogen to
the appearance of neoplastic transforma-
tion of hamster embryo cells in vitro.

Since TPA can alter the morphology of
human, mouse and chick cells (Sivak, et al.,
1969; Diamond et al., 1974) we studied the
effects of TPA on the transformation of
hamster cells, as measured by the presence
of foci of piled up cells in the monolayer
cultures. Subsequently, as a measure of
their malignancy, we investigated the
ability of these cells to grow in soft agar
and induce tumours in animals.

TWO-STAGE MALIGNANT TRANSFORMATION

MATERIALS AND METHODS

Cells.-Primary cell pools 1839, 583 and
241 were prepared from random-bred Syrian
golden hamster embryos (ELA/ENG) (Engle
Laboratory Animals, Farmersburg, IN) at
13 days of gestation. The cells were planted at
2 x 107/150 cm2 flask (Costar Products, Cam-
bridge, MA) in Dulbecco's Modified Eagle's
Medium (DMEM) (Grand Island Biological
Corp., Grand Island, NY) supplemented with
2 mm L-glutamine and 10% heat-inactivated
foetal calf serum (Reheis Chemical Co.,
Phoenix, AZ). Only serum determined by the
Viral   Resources  Laboratory   (FCRC,
Frederick, MD) to be free from mycoplasma
and bacteriophage was used. All cell cultures
were fed 3 times per week. Cells were dis-
aggregated with ENZAR T (Reheis) when
90 % confluent.

Chemicals.-Stock solutions of 3-methyl-
cholanthrene (MCA) and dibenz(a,h)anthra-
cene (DBA) (Eastman Kodak, Rochester,
NY) and TPA (Consolidated Midland Corp.,
Brewster, NY) were prepared in dimethyl-
sulphoxide (DMSO) (Crown Zellerbach Corp.,
Camas, WA). The chemicals were further
diluted in DMEM to obtain the required dose
in a final concentration of 0.2% DMSO.

Simultaneous treatment with MCA and
TPA.-Cells from culture 1839 were seeded
at 5 x 106/75 cm2 flask 4 h before chemical
treatment with either DMEN, DMEN+0-2%
DMSO, DMEM+0-5 ,Lg/ml MCA, DMEM+
01 ,ug/ml TPA or the combination TPA+
MCA. Cultures were maintained at 37?C in a
humidified incubator in an atmosphere
of 10% CO2 in air. Subcultures were made by
dividing the cells 1: 2 and 1: 4. Cultures
reaching confluence after a 1: 2 subdivision
were considered to have undergone one popu-
lation doubling (PD), 2PD after a 1: 4 sub-
division, and 4 PD after a 1:16 subdivision.
Cells were exposed to both carcinogen and
TPA continuously for 30 days. Thereafter, all
cultures were maintained in DMEM alone.

Simultaneous treatment with MCA and
varying doses of TPA.-Following the same
procedure as described above, cell culture 583
was treated with DMEM, DMEM+0-2%
DMSO, 0-01, 0-1, or 1 jug/ml of TPA alone
or in combination with MCA. Chemical treat-
ment was continued for 30 days, followed by
maintenance on DMEM alone.

Carcinogen treatment followed by TPA treat-
ment.-Cells from culture 241 were seeded at

5 x 106/75 cm2 flask and after 4 h treated for
48 h as follows: (1) DMEM, (2) DMEM+0-2%
DMSO, (3) 05 ,ug/ml MCA and (4) 1-0 ,ug/ml
DBA. On Day 2 the cultures were re-fed with
DMEM and allowed to grow to confluence
(Day 6). They were then subcultured into 2
groups. Twenty-four hours after subculture
(Day 7) one flask from each group was treated
with 0-1 ,ug/ml TPA and the other re-fed with
DMEM, producing 8 groups. At subculture,
2 flasks were made for each treatment regime.
No toxicity was seen in any of the cultures,
and all cultures reached confluence within
48 h of a 1:2 subdivision. Therefore, split
ratios were increased to a level which allowed
all cultures to become confluent within 7 days.
The population doubling level was adjusted
according to the subculture ratio 1:16. On
Day 30, half of each group of flasks which had
been treated with TPA were re-fed with
DMEM and remained on DMEM for the rest
of the experiment. At the same time (Day 30),
half of each group of flasks which had re-
ceived only the 48 h chemical were treated
with TPA. Treatment was continued through-
out the rest of the experiment. The remaining
flasks in this group were never exposed to
TPA. This produced 16 groups.

Transformation.-Morphological transfor-
mation was defined as an increase in cell
density accompanied by numerous cells
growing suspended in the medium as well as
on the surface of the cell sheet forming foci,
with a loss of polar orientation of the cells on
the monolayer. After morphological trans-
formation was seen, 105 cells from each culture
were seeded in soft agar (MacPherson, 1969)
and 106 cells were injected s.c. into 9-day-old
suckling hamsters. Soft agar cultures were
held for 45 days unless macroscopic colonies
were seen earlier. Inoculated hamsters were
held for 90 days before being assessed as
negative for tumour formation.

RESULTS

Effects of simultaneous treatment with MCA
and TPA

Cells from culture 1839 which were
treated with MCA or MCA+0-1 ,ug/ml
TPA showed morphological transforma-
tion by Day 30. At PD 20, the cells were
plated into soft agar and injected into
suckling hamsters. There was an increase

9

J. A. POILEY, R. RAINERI AND R. J. PIENTA

TABLE I.-Effects of simultaneous treatment with 12-0-tetradecanoyl-phorbol-13-acetate

(TPA) and 3-methylcholanthrene (MCA) on transformation of hamster embryo cells

Treatment

(2 flasks)
DMEMt
DMSO

TPA (01 ,g/rnl)
MCA (0 5 /ug/ml)
MCA+TPA

Growth in soft

agar (%)
Morphological ,         A

transformation  PD20         PD40
None                t          ND
None              -            ND
None?                          ND

(PD9) 30 days   3 (7 days)     3 (7 days)
(PD9) 30 days     -            2 (7 days)

Terminal PD

20
20
34
>95
>95

Tumorigenicity*
PD20 (latency)
0/4
0/6
0/9

6/6 (7 days)

5/7 (20 days)

* Number of hamsters with tumours/number inoculated; all tumours anaplastic spindle-cell sarcomas.
t Abbreviations used: DMEM =Medium control; DMSO = Solvent control; ND = Not determined (cultures
had terminated); PD =Population Doubling.

t Negatives maintained in culture for 45 days.

? Retained the rapid growth rate, orientation and characteristics of earlier-passage control cells.

in the latent period to tumour formation,
and a slightly lower incidence of tumour
induction, in hamsters receiving (MCA+
0-1 ug/ml TPA)-treated cells compared
with those receiving cells treated with
MCA alone (Table I). No tumours were
seen in hamsters receiving cells from
cultures treated with DMEM, DMSO, or
0.1 ,tg/ml TPA alone. Only cells from
those cultures treated with MCA alone
produced rapidly growing colonies in soft
agar.

Effects of simultaneous treatment with MCA
and varying doses of TPA

Culture 583 treated with MCA or MCA+
TPA (at any of the doses used) showed
morphological transformation, whilst con-
trol cultures, or those receiving TPA alone,
did not (Table II). (This cell culture was
characterized by a longer latent period to
morphological transformation than culture
1839). At PD 20, the cells were plated in
soft agar and injected into suckling ham-
sters. Culture 583 responded similarly to
culture 1839 when treated with MCA or
MCA+0-1 ,ug/ml TPA. When      it was
treated with MCA+-0-01 or 1 ,ug/ml TPA;
or with TPA alone, the cells failed to induce
tumours in animals or grow in soft agar.
This was also true of untreated controls. All
cultures which had not reached senescence
at PD 40 were retested for growth in soft
agar and tumorigenicity. Cultures treated
with MCA, MCA+0-01 or 0-1 jug/ml TPA

grew in soft agar and produced tumours in
hamsters. The only remaining non-
tumourigenic culture which had not
reached senescence (MCA+1 jug/ml TPA)
grew in soft agar (<0.1%) and produced
tumours in animals when it was retested
at PD 64.

Effects of carcinogen treatment followed by
TPA treatment

Of the 16 different treatment regimens,
only 4 produced malignantly transformed
cells which grew in soft agar and produced
tumours in animals. These were from cells
treated with (1) MCA for 48 h, (2) MCA
for 48 h followed by TPA at Day 30, (3)
DBA for 48 h followed by TPA at Days 7-
30 and (4) DBA for 48 h followed by TPA
at Day 30 (Table III). As in the 2
studies mentioned above, no transforma-
tion occurred when cells were treated with
DMEM,     DMEM+0.2%      DMSO     or
DMEM+0-1 pg/ml TPA. All the cultures
treated with TPA at different times after
treatment with DMEM or DMEM+0-2%
DMSO responded similarly, and one
representative group is included in the
table. Cells which transformed when
treated with MCA followed by TPA at
Day 30 had a slightly shorter latency than
those transformed by MCA alone. Simi-
larly, cultures which transformed after
treatment with DBA followed on Day 30
by TPA had a shorter latency than those
transformed after treatment with DBA

10

TWO-STAGE MALIGNANT TRANSFORMATION

OD

0

_

V

V

_a        *H

PI)        )

*H
.,1

?          0

0

00

0J2

p~~~~~~

"02

t~~~t 00 -C,

co   tr -  -
-Q  Q  Z ? Z rb

9                              Co

o ;          P4 aq -I m m ?-- = = =

$-,                   ~~~~~~~~~~~~~0)

0         -              AAA        A   0

-~~~~~~~~~~~~ U)

_~~~~~~~~~
"I                                   I _

CO

1o X
; .t

o

\

.-    0

*. rn

Z  0

S * t -=

CO                  *

0  s

12

~~~~~~~~~~~~~~~ -
P~~  ZZZZZc~~~c-  I

o

4 1 T   i i > i i  I  5

0)                  0

1.0~~~~~~

t   O  U   Q  Q  Q  Q   O  O  O~~~~~~~~COCl

0    P14 P-1  "0  0 4
12 ~ ~ ~ ~ ~ ~ 1

o            - ;

;   So? o_o++ +  E S

O     ??*

PL4 P., P-p4

Er-I  E--, E--q E--q ~ ~

11

J. A. POILEY, R. RAINERI AND R. J. PIENTA

m
t-

ci2

"0

ho

&Q m

_      "

10 00

3  00C043  --  -  -  --  -  N-

eo  eo  1 104 _4  q  14  -00  -  P I

C-

H      A A A A   A A A

0

EH~~~~~~~~~~~~i cz

100o  C   N N oo N  0-   0  "0
COOC  01  0  0 100 10 1  II  +Z.

I

0 0

4 H

" q   0 "0q   " 0 "

Q   g  0  p  0 C   10  1  10

0   0   0   0 0   CO  o 0 1

"0  0
0 t-  0  M  o t-  m c3

C4.4 ~ ~ ~ ~ ~ Q >,  3

- ? n

0  o        "  S

0   P.,00 0   0  0 0 0 0

_      Z           .  0 ZN  N  N c   e

tb   3 S   . 52h   *5 C+2.  2

0                    4? C   oS O;  -O

P  ~ ~ 00       e~
co  QQo Z    o

_~~~~~~~~b I c3

0~ ~ ~ ~    F F

12

*

-4-

>

q

CD

*D CD

0

4QO
V

0t

?

I.

ll?l
I

TWO-STAGE MALIGNANT TRANSFORMATION

followed by TPA on Days 7-30. Neither of
the cultures treated with MCA or DBA
and followed by continuous treatment
with TPA from Day 7 grew in soft agar
or produced tumours in hamsters, although
a terminal PD was not reached (> 95
PD's).

DISCUSSION

Using hamster embryo cells, we studied
the effect of TPA treatment on malignant
transformation. Parameters measured
included: morphological transformation,
increased lifespan, growth in soft agar and
induction of tumours in animals. Our
studies show that TPA can act either as an
inhibitor or a promoter of malignant trans-
formation in hamster embryo cells depend-
ing on the time interval between treatment
with carcinogen and the administration of
TPA. Similarly, TPA-dependent stimula-
tion or inhibition of the rate of focal trans-
formation of mouse fibroblasts (C3H/10T
1/2) cells was shown to depend upon the
length of time between carcinogen and
TPA treatments (Mondal et al., 1976).
When the hamster cells were treated with
MCA or MCA+TPA, morphological trans-
formation was observed at the same time
irrespective of treatment. Similarly, all
the groups showing morphological trans-
formation grew beyond the lifespan of the
control cultures. However, induction of
tumours in animals did not occur until 20
PD later with the lowest TPA concentra-
tion, and 44 PD later with the highest
TPA concentration. Similar observations
were made concerning growth in soft agar.
When TPA was administered simul-
taneously with MCA, criteria indicative of
malignant transformation were inhibited.

Treatment with TPA 7 days after MCA
produced complete inhibition of both
tumour formation and growth in soft agar.
In contrast to our observations, others
have shown that when TPA was applied
3-5 days after carcinogen treatment in
mouse cells (Mondal et al., 1976) or 7 days
after carcinogen treatment in rat cells
(Lasne et al., 1977) enhanced rates of focal
transformation occurred.

Treatment with TPA 27 days after MCA
treatment reduced the latent period to
morphological transformation and in-
creased the tumour incidence. Perhaps a
longer time is required to complete the
first stage of transformation in hamster
than in mouse (Mondal et al., 1976) or rat
cells (Lasne et al., 1977). Treatment with
TPA simultaneously with, or soon after,
treatment with a transforming dose of
MCA may prevent the repair of carcinogen
damage to the cells.

A promoting effect was found when
hamster embryo cells were treated with a
subtransforming dose of DBA and TPA
was administered either between 7 and 27
days, or from 27 days after DBA treat-
ment. However, in all experiments with a
transforming dose of carcinogen, the con-
tinued presence of TPA in the culture
medium during the first 30 days appeared
to have inhibited the neoplastic trans-
formation of the cells. Cells acquired the
properties of growth in soft agar and
tumour production in animals only after
TPA had been removed, or when treat-
ment with TPA did not begin until 27
days after treatment with a transforming
dose of MCA.

Morphological changes appeared to
reflect the initial events in transformation
that are followed later by tumorigenesis
and growth in soft agar. The studies re-
ported here indicate that cells become
tumorigenic before they acquire the
ability to grow in soft agar (Tables I and
II). This confirms the use of the generally
observed in vitro characteristic of growth
in soft agar as a valid indicator of the
tumorigenic potential of cells. The inhibi-
tory effects of TPA on characteristics of
transformation may also occur through an
inhibition of the mechanisms responsible
for invasive properties of the transformed
cells. The use of TPA in combination with
MCA or DBA on hamster embryo cells to
delay the appearance of malignant charac-
teristics provides a model system for
studying the sequence of events from
morphological transformation to tumouri-
genieity.

13

14              J. A. POILEY, R. RAINERI AND R. J. PIENTA

Research sponsored by the National Cancer Insti-
tute under contract No NOI-CO-75380, with Litton
Bionetics, Inc.

Appreciation is extended to Dorothy Cavanaugh
for her expert technical assistance.

REFERENCES

BOUTWELL, R. K. (1964) Some biological aspects of

skin carcinogenesis. Prog. Exp. Tumor Res., 4, 207.
BOUTWELL, R. K. (1 974) The function and mechanism

of promoters of carcinogenesis. CRC Crit. Rev.
Toxicol., 2, 419.

DIAMOND, L., O'BRIEN, S., DONALDSON, C. &

SHIMIZU, Y. (1974) Growth stimulation of human
diploid fibroblasts by the tumor promoter, 12-0-
tetradecanoyl-phorbol- 13-acetate. Int. J. Cancer,
13, 721.

LASNE, C., GENTIL, A. & CHOUROULINKOV, I. (1974)

Two-stage malignant transformation of rat
fibroblasts in tissue culture. Nature, 247, 490.

LASNE, C., GENTIL, A. & CHOUROULINKOV, I. (1977)

Two-stage carcinogenesis with rat embryo cells in
tissue culture. Br. J. Cancer, 35, 722.

MACPHERSON, I. (1969) Agar suspension culture for

quantitation of transformed cells. In Fundamental
Techniques in Virology. Eds K. Abel and N. P.
Salzman. New York: Academic Press. p. 214.

MONDAL, S. & HEIDELBERGER, C. (1976) Transfor-

mation of C3H/1OT1/2C18 mouse embryo-
fibroblasts by ultraviolet irradiation and a phorbol
ester. Nature, 260, 710.

MONDAL, S., BRANKOW, D. W. & HEIDELBERGER, C.

(1976) Two-stage chemical oncogenesis in cultures
of C3H/10T1/2 Cells. Cancer Res., 36, 2254.

SIVAK, A. & VAN DUUREN, B. L. (1970) RNA

synthesis induction in cell culture by a tumor
promoter. Cancer Res., 30, 1203.

SIVAK, A., MASSMAN, B. T. & VAN DUUREN, B. L.

(1972) Activation of cell membrane enzymes in
the stimulation of cell division. Biochem. Biophys.
Res. Commun., 46, 605.

SIVAK, A., RAY, F. & VAN DUUREN, B. L. (1969)

Phorbol ester tumor-promoting agents and
membrane stability. Cancer Res., 29, 624.

TROSKO, J. E., YAGER, J. D. JR., BOWDEN, J. T. &

BUTCHER, F. R. (1975) The effects of several
croton oil constituents on two types of DNA
repair and cyclic nucleotide levels in mammalian
cells in vitro. Chem-Biol. Interact., 11, 191.

				


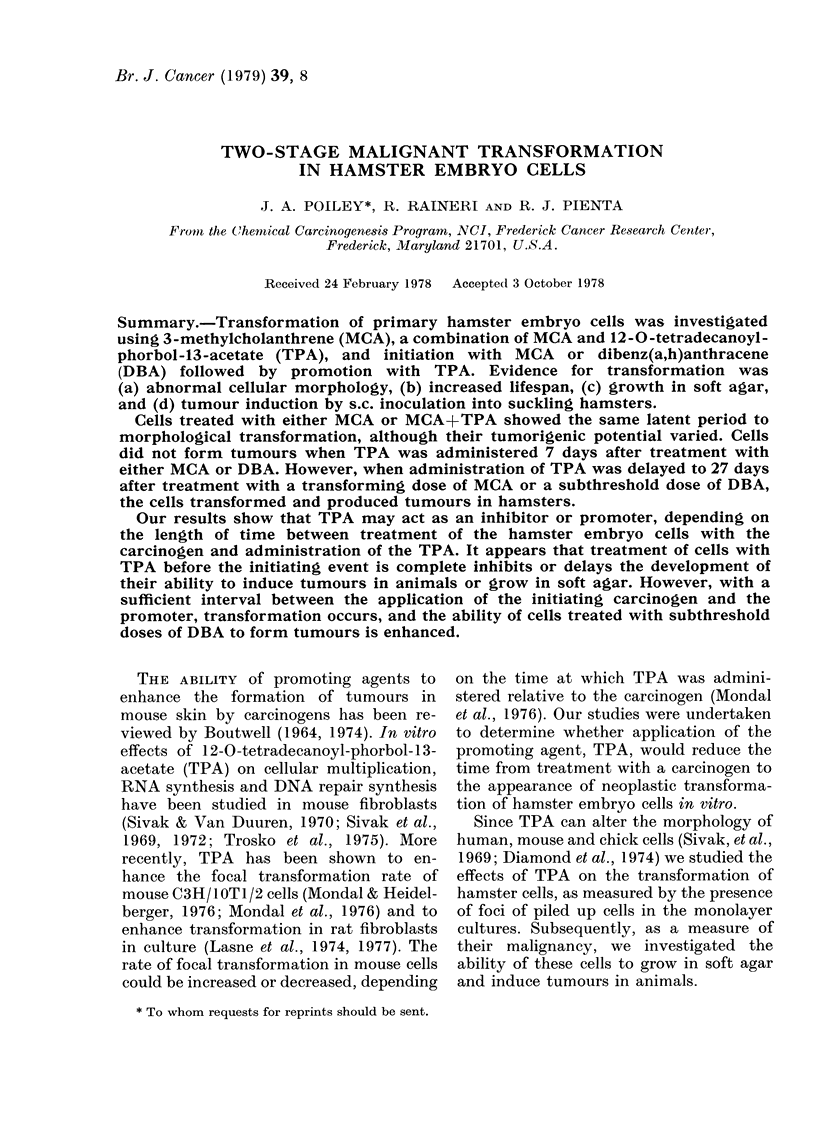

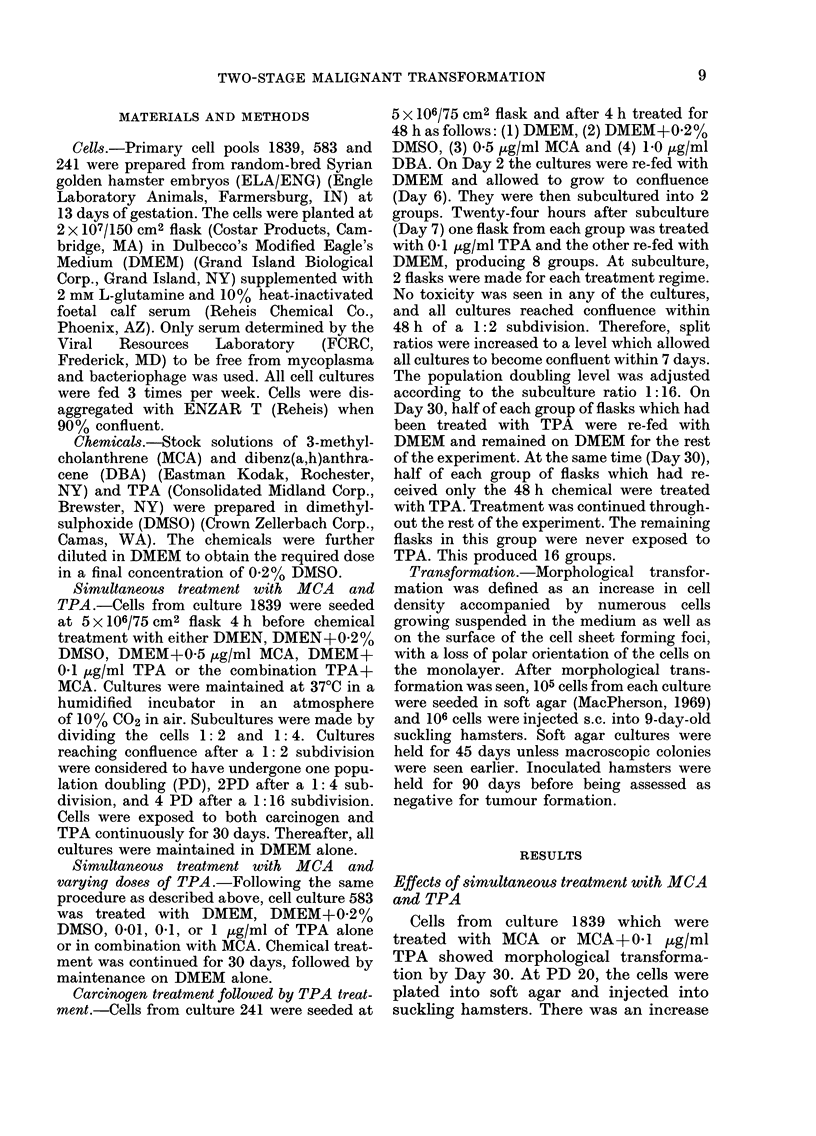

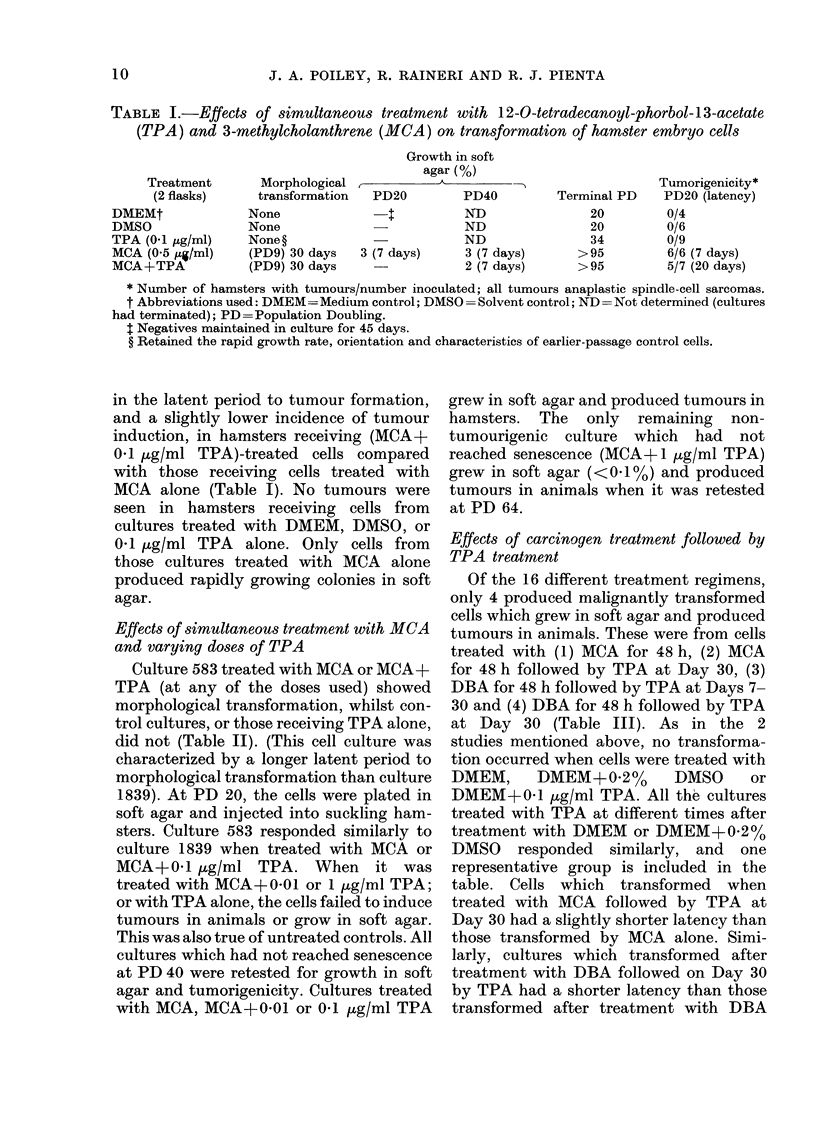

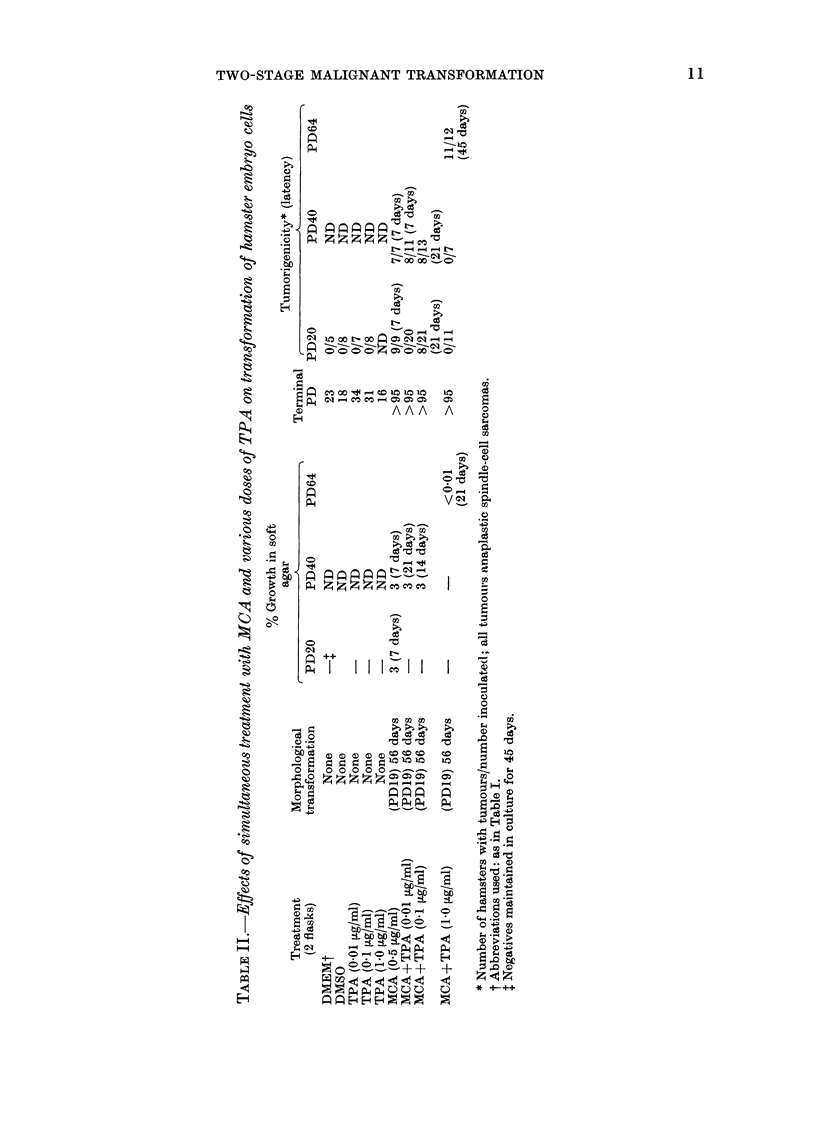

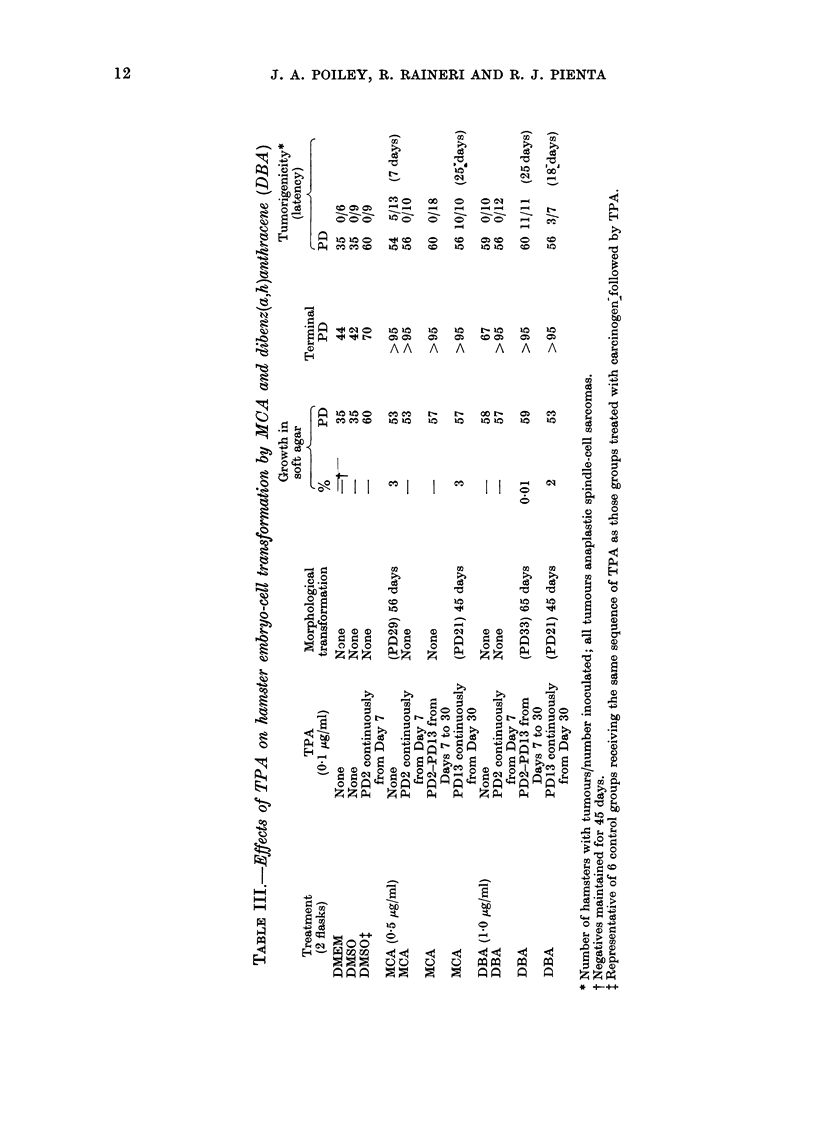

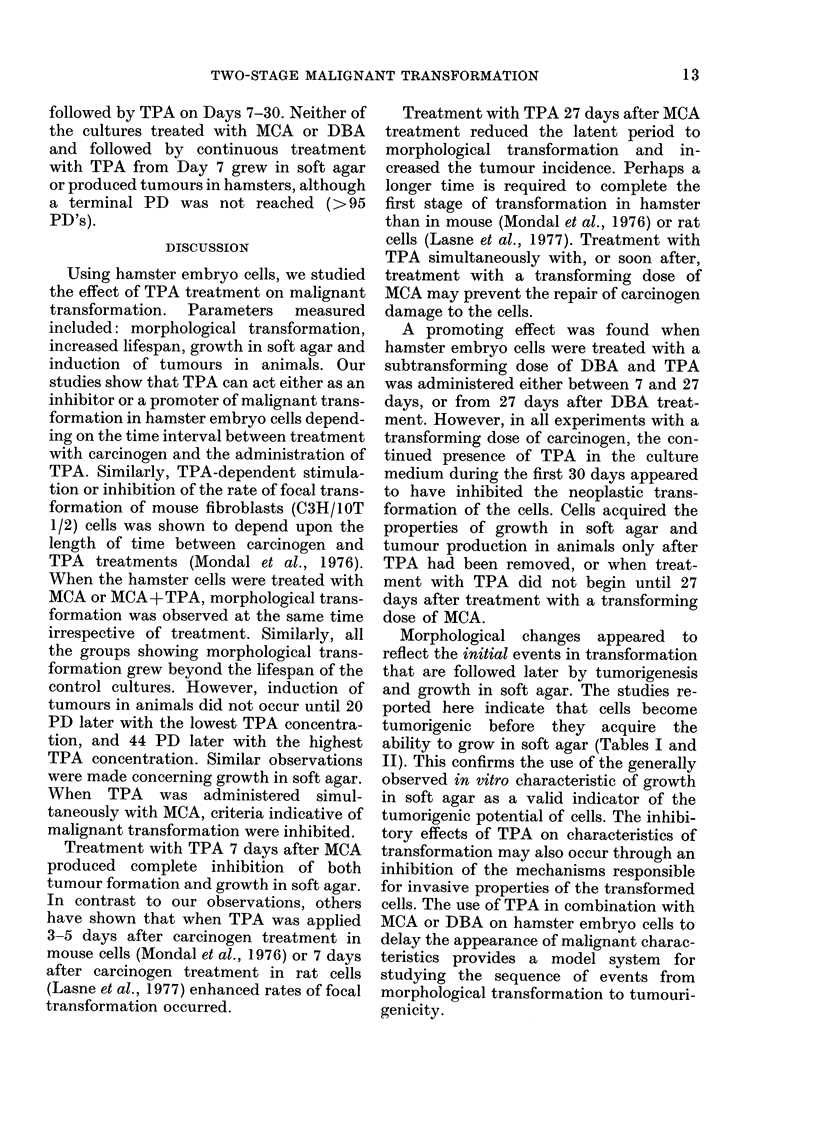

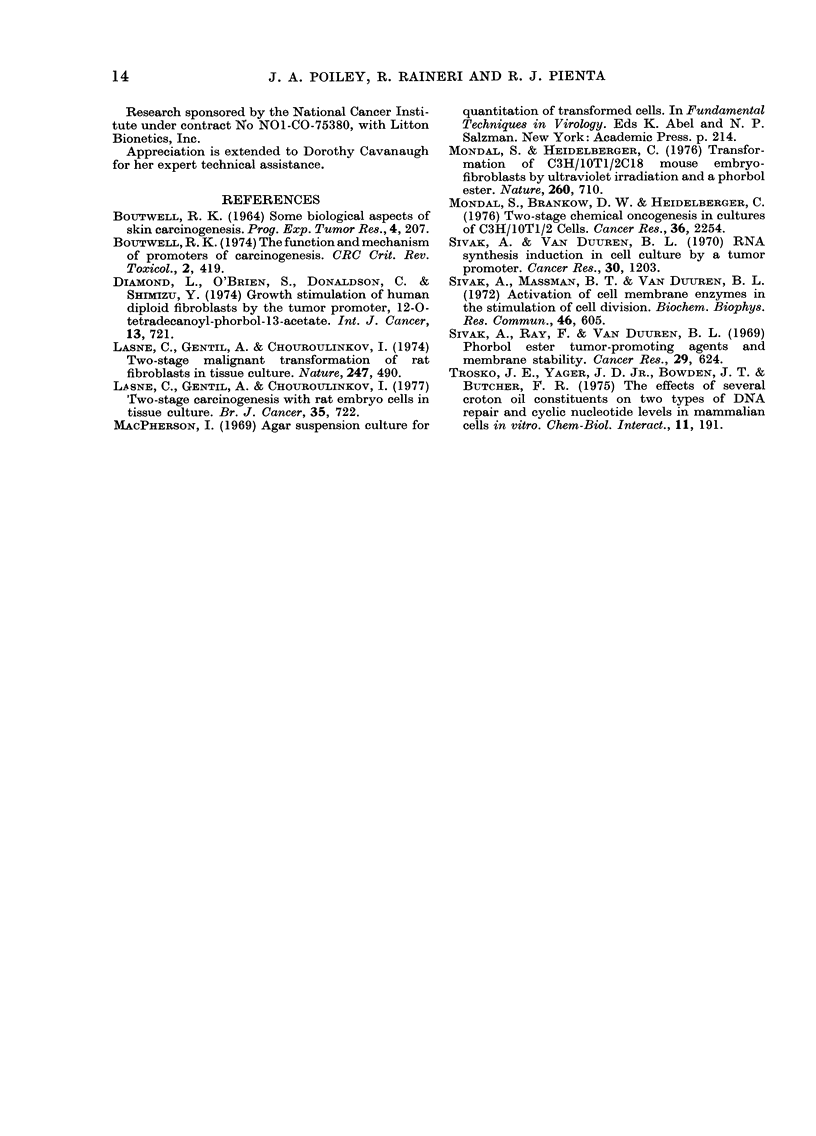

